# Effect of balloon pre-dilation on performance of self-expandable nitinol stent in femoropopliteal artery

**DOI:** 10.1007/s10237-022-01641-x

**Published:** 2022-10-25

**Authors:** Ran He, Liguo Zhao, Vadim V. Silberschmidt

**Affiliations:** 1 Wolfson School of Mechanical, Electrical and Manufacturing Engineering, Loughborough University, Epinal Way, Loughborough, LE11 3TU UK; 2 College of Energy and Power Engineering, Nanjing University of Aeronautics and Astronautics, Nanjing, 210016 People’s Republic of China

**Keywords:** Balloon pre-dilation, Nitinol stent, Fatigue resistance, Arterial damage, In-stent restenosis, Finite element analysis

## Abstract

Balloon pre-dilation is usually performed before implantation of a nitinol stent in a femoropopliteal artery in a case of severe blockage or calcified plaque. However, its effect on performance of the nitinol stent in a diseased femoropopliteal artery has not been studied yet. This study compares the outcomes of stenting with pre-dilation and without it by modelling the entire processes of stent deployment. Fatigue deformation of the implanted stent is also modelled under diastolic–systolic blood pressure, repetitive bending, torsion, axial compression and their combination. Reduced level of stress in the stent occurs after stenting with pre-dilation, but causing the increased damage in the media layer, i.e. the middle layer of the arterial wall. Generally, pre-dilation increases the risk of nitinol stent’s fatigue failure. Additionally, the development of in-stent restenosis is predicted based on the stenting-induced tissue damage in the media layer, and no severe mechanical irritation is induced to the media layer by pre-dilation, stent deployment or fatigue loading.

## Introduction

Stenting is one of the minimally invasive treatments for peripheral artery disease, by expanding and keeping the diseased artery open. Pre-dilation should be performed prior to stenting when there is significant stenosis, occlusion or the stent cannot be properly positioned (including moderate stenosis), aiming to assist with stent placement and vessel expansion. However, pre-dilation induces additional mechanical stretching to the vessel, which increases the arterial damage (He et al. 2020a) and, consequently, the risk of in-stent restenosis (ISR) (i.e. a reoccurrence of stenosis after stenting; He et al. 2020c).

Some modelling work was carried out to study the effect of pre-dilation on the stent performance and arterial damage. Conway et al. (2017) investigated the effects of pre-dilation on the artery’s mechanical response to stent deployment, with consideration of the Mullins effect (stress softening) and plastic deformation of the plaque. Their results indicated that direct stenting without pre-dilation resulted in slightly higher stresses than that with pre-dilation, for all damage models used for the plaque. The effect of pre-dilation on the performance of a bioresorbable polymeric stent was also investigated, with a focus on assessing the tissue damage in the artery and the plaque caused by a percutaneous coronary intervention (PCI) procedure (He et al. 2020a). The results showed that the pre-dilation softens the artery and the plaque, making the diseased artery easier to expand in stenting.

Thanks to their unique superelastic behaviour, self-expandable nitinol stents have excellent flexibility and the ability to recover from deformation. As a result, nitinol stents are particularly attractive for treating blocked femoropopliteal arteries which are under complex loading conditions caused by daily physical activities of patients. Compared to conventional angioplasty, superelastic nitinol stents provide favourable safety and durability (Vogel et al. 2003; Mewissen 2004; Duda et al. 2006), and demonstrate superior clinical outcomes (Schillinger et al. 2006; Laird et al. 2010; Fusaro et al. 2013). However, normal physical activities exert repetitive external forces, including physiological and biomechanical fatigue loadings, on the implanted nitinol stents, leading to their fracture (MacTaggart et al. 2014). Clinical studies reported a significantly increased rate of nitinol stent fracture with the increase of post-implantation time. For instance, Schlager et al. (2005) reported that the fracture rate was 2% for Dynalink/Absolute stents after a mean 15 ± 9 months, but increased to 28% after mean 32 ± 16 months. Bosiers et al. (2009) carried out a prospective, multicentre, non-randomized study enrolled 151 undergoing percutaneous treatment of de novo, restenotic or reoccluded superficial femoral artery lesions, with a 1-year stent fracture rate of 8.1%. Also, stent fracture is found highly associated with restenosis, thrombosis, pseudo-aneurysm or embolisation (Reis et al. 2019; Kim et al. 2020). Hence, improving the fatigue resistance of stents has become one of the main objectives for stent design and manufacturing.

Fatigue behaviour of nitinol stents was studied by modelling approaches. For instance, Harvey (2011) modelled an artery based on CT imaging to investigate the fatigue performance of a stent under pulsatile and articulation loads by using finite element (FE) method. Advanced nonlinear FE method showed its feasibility for fatigue prediction for nitinol stents in human arteries. A series of numerical studies on the risk of fatigue failure of nitinol stents were carried out by a research team at Politecnico di Milano (Italy), with a focus on the effects of failure criteria, loading conditions and plaque morphology (Petrini et al. 2012, 2016; Meoli et al. 2013, 2014; Dordoni et al. 2014; Allegretti et al. 2018). Lei et al. (2019) showed both atherosclerotic plaque and physiological loading conditions should be considered to accurately assess the fatigue behaviours of stents. A computational study was also carried out to investigate the fatigue resistance of nitinol stent subjected to walk-induced motion of a femoropopliteal artery (He et al. 2021a). The results demonstrated that the pulsatile blood pressure did not contribute much to stent’s fatigue failure, and the combined loadings imposed the highest risk to stent’s fatigue failure, with the main contribution by bending, followed by axial compression and torsion. However, no studies investigated the effect of pre-dilation on the fatigue failure risk of an implanted nitinol stent, although pre-dilation is frequently performed before the implantation of nitinol stents.

In the PCI procedure, the stent expands the blood vessel, which causes damage to the media layer, and thus activates the leucocyte transmigrating into the vessel wall. Triggered by this process, the smooth muscle cells in the media layer proliferate and then migrate to form neointima, the major contribution of ISR (Hoffmann and Mintz 2000). The effect of stent design on the development of ISR in the coronary artery was investigated by modelling work as well in Lally and Prendergast (2006), however, with critical limitations. They did not consider plaque in their model and assumed the arterial wall was isotropic hyperelastic. Recently, a positive correlation between the development of ISR and the stenting-induced tissue damage in the media layer has been established based on models of damage and growth of tissues (He et al. 2020c), which was subsequently used to predict the development of ISR after deployment of a single stent and three stents with and without overlap (He et al. 2020b). The prediction showed that the single stent and non-overlapping stents induced the lowest and highest rates of ISR, respectively. However, the study of ISR for the nitinol stent has not been attempted yet.

Therefore, the aim of this study was to investigate the effect of pre-dilation on the deployment of the nitinol stent in an atherosclerotic femoropopliteal artery and the subsequent fatigue resistance of the stent subjected to walk-induced motion of the artery, using advanced FE analysis. The developed FE models simulated a diseased artery with a layered structure and a self-expandable nitinol stent, all described with advanced constitutive relationships. The study was implemented by modelling the processes of stent crimping, self-expansion in the diseased artery and deformation under diastolic–systolic blood pressure, repetitive bending, torsion and axial compression as well as their combination, with pre-dilation and without it. The lumen gain, stresses in the stent and the artery and arterial damage after stenting were assessed and evaluated as the outcomes of nitinol stent implantation, with the cyclic stresses in the nitinol stent used to evaluate the fatigue behaviour. In addition, the development of ISR after stenting was predicted, based on the assessment of tissue damage.

## Constitutive models

### Ogden model with damage for plaque

**Fig. 1:**
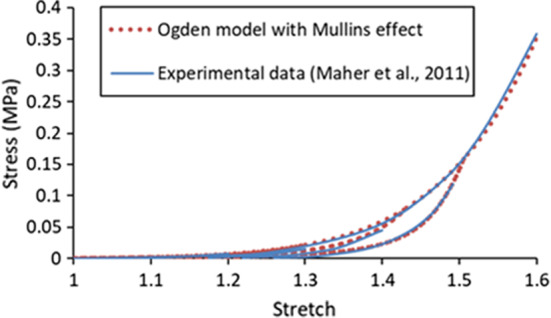
Stress–stretch response of plaque simulated using Ogden model with Mullins effect in comparison with experimental data in Maher et al. (2011) (unloading occurred at stretch levels of about 1.2, 1.3, 1.4 and 1.5)

The constitutive behaviour of the plaque was assumed to be isotropic with stress softening and described by the first-order Ogden hyperelastic model (Ogden 1972) with Mullins effect (Ogden and Roxburgh 1999). The experimental stress–stretch data obtained in Maher et al. (2011) for plaque were used to determine the values of model parameters by MCalibration (PolymerFEM, LLC), given in Table 1, where *ρ* is the density of the plaque (Rahdert et al. 1999). The simulated stress–stretch response for the plaque under uniaxial tension is shown in Fig. 1, which is in a good agreement with the experimental results (Maher et al. 2011).

**Table 1:** Values of parameters of Ogden model with Mullins effect for plaque

*ρ* (t/mm^3^)	$\mu$(MPa)	$\alpha$	$D$(MPa^−1^)	*r*	*m* (mJ/mm^3^)
1.22E-9	0.00397	13.837	0.239	1.3	0.008

### Modified HGO-C model for arterial layers

The anisotropic hyperelastic constitutive behaviour of arterial layers was described by the modified HGO-C model (Nolan et al. 2014) with damage (Fereidoonnezhad et al. 2016), with a pseudo-energy potential given by

1 $$\begin{array}{c}\psi ={\psi }_{\mathrm{vol}}+{\overline{\psi }}_{m}^{0}+\sum_{\alpha =1}^{N}\left[{\eta }_{f,\alpha }{\psi }_{f,\alpha }^{0}+{\phi }_{f,\alpha }\left({\eta }_{f,\alpha }\right)\right]\\-\left[\left(1-{\eta }_{\mathrm{in}}\right){\psi }_{\mathrm{in}}^{*}\left({I}_{i}^{*}\right)+{\phi }_{\mathrm{in}}\left({\eta }_{\mathrm{in}}\right)\right],\end{array}$$

2 $$\begin{array}{c}{\psi }_{\mathrm{vol}}=\frac{1}{D}\left(\frac{{J}^{2}-1}{2}-\mathrm{ln}J\right),\end{array}$$

3 $$\begin{array}{c}{\overline{\psi }}_{m}^{0}={C}_{10}\left({\overline{I} }_{1}-3\right),\end{array}$$

4 $$\begin{array}{c}{\psi }_{f,\alpha }^{0}=\frac{{k}_{1}}{2{k}_{2}}\sum_{\alpha =1}^{N}\left[\mathrm{exp}\left({k}_{2}{\langle \kappa \left({\overline{I} }_{1}-3\right)+\left(1-3\kappa \right)\left[{I}_{4\left(\alpha \alpha \right)}-1\right]\rangle }^{2}\right)-1\right],\end{array}$$

where ${\overline{\psi }}_{m}$ and ${\psi }_{f,\alpha }^{0}$ are the isochoric energies stored in the non-collagenous matrix and collagen fibres, respectively, ${\phi }_{f,\alpha }\left({\eta }_{f,\alpha }\right)$ and ${\phi }_{\mathrm{in}}\left({\eta }_{\mathrm{in}}\right)$ are the damage functions for the Mullins effect and permanent deformation, respectively, ${C}_{10}$ and ${k}_{1}$ are the stress-like parameters, ${k}_{2}$ is the dimensionless parameter, $\langle \rangle$ stands for the Macaulay brackets, $\kappa (0\le \kappa \le 1/3)$ is the temperature-dependent material parameter describing the level of dispersion in the fibre directions, $N$ is the number of families of fibres ($N\le 3$), ${I}_{1}$ is the first principal invariant of the right Cauchy–Green deformation tensor $\mathbf{C}$ (i.e. ${I}_{1}=\mathrm{tr}\mathbf{C}={\lambda }_{1}^{2}+{\lambda }_{2}^{2}+{\lambda }_{3}^{2}$ and its isochoric part is ${\overline{I} }_{1}={J}^{-2/3}{I}_{1}$), and ${I}_{4(\alpha \alpha )}$ are the invariants of $\mathbf{C}$ and ${\mathbf{a}}_{\alpha }$ (i.e. ${I}_{4(\alpha \alpha )}={\mathbf{a}}_{\alpha }\cdot \mathbf{C}{\mathbf{a}}_{\alpha }$, with ${\mathbf{a}}_{\alpha }$ being the unit vectors used to define the mean directions of the fibres in the reference configuration). In addition, ${\eta }_{f,\alpha }$ and ${\eta }_{\mathrm{in}}$ are the damage variables for the Mullins effect and permanent deformation, respectively, and ${\psi }_{\mathrm{in}}^{*}\left({I}_{i}^{*}\right)$ is the (anisotropic) inelastic energy dissipation, which are given by

5 $$\begin{array}{c}{\eta }_{f,\alpha }=1-\frac{1}{{r}_{f}}\mathrm{erf}\left[\frac{1}{{m}_{f}}\left({\psi }_{f,\alpha }^{\mathrm{max}}-{\psi }_{f,\alpha }^{0}\right)\right],\end{array}$$

6 $$\begin{array}{c}{\eta }_{\mathrm{in}}=\frac{\mathrm{tanh}{\left[\frac{{\overline{\psi }}_{m}^{0}+{\psi }_{f,\alpha }^{0}}{{\left({\overline{\psi }}_{m}+{\psi }_{f,\alpha }\right)}^{\mathrm{max}}}\right]}^{{m}_{2}}}{\mathrm{tanh}1},\end{array}$$

7 $$\begin{array}{c}{\psi }_{\mathrm{in}}^{*}\left({I}_{i}^{*}\right)={C}_{10}^{*}\left({\overline{I} }_{1}^{*}-3\right)+\frac{{k}_{1}^{*}}{2{k}_{2}^{*}}\sum_{\alpha =1}^{\mathrm{N}}\left[\mathrm{exp}\left({k}_{2}^{*}{\left\langle {\kappa }^{*}\left({\overline{I} }_{1}^{*}-3\right)+\left(1-3{\kappa }^{*}\right)\left[{I}_{4\left(\alpha \alpha \right)}^{*}-1\right]\right\rangle }^{2}\right)-1\right],\end{array}$$

where ${m}_{2}$, ${C}_{10}^{*}$, ${k}_{1}^{*}$, ${k}_{2}^{*}$ and ${\kappa }^{*}$ are the material parameters for permanent deformation, and ${\overline{I} }_{1}^{*}$ and ${I}_{4\left(\alpha \alpha \right)}^{*}$ are the strain invariants at the peak deformation of the loading history (i.e. when ${\overline{\psi }}_{m}^{0}+{\psi }_{f,\alpha }^{0}={\left({\overline{\psi }}_{m}+{\psi }_{f,\alpha }\right)}^{\mathrm{max}}$). Here, it is noted that volumetric deformation does not affect the damage (i.e. the damage variables are independent of the volumetric terms according to Eq. (1)).

**Fig. 2:**
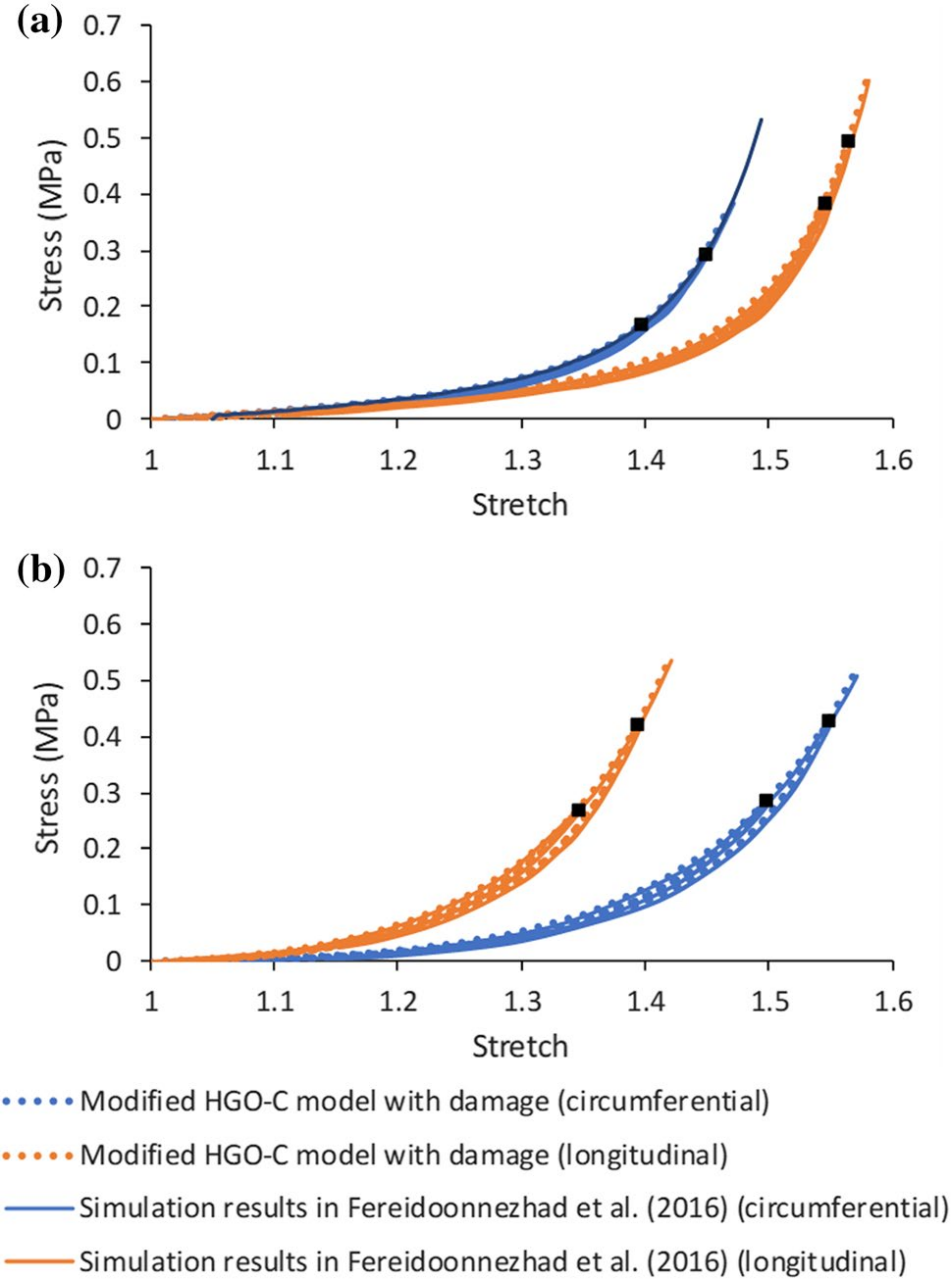
Stress–stretch responses of **a** media and **b** adventitia layers, simulated using modified HGO-C model with damage, in comparison with those in Fereidoonnezhad et al. (2016; black squares indicate unloading points)

The model parameters were provided in Fereidoonnezhad et al. (2016) based on fitting the experimental stress–stretch data (Weisbecker et al. 2012). In this study, two families of fibres were assumed to be embedded symmetrically in the tangential surface of each arterial layer (no components in the radial direction). The angle between the mean direction of fibres and the circumferential direction in the artery was represented by $\varphi$. A VUMAT subroutine was written for the modified HGO-C model with damage to be implemented in Abaqus. The simulated stress–stretch responses for the arterial layers under uniaxial tension are shown in Fig. 2a and b, demonstrating a good agreement with the corresponding results in Fereidoonnezhad et al. (2016). Although aorta’s data were used in this study, the mechanical responses of the arterial layers fell in the range for femoropopliteal arteries (He et al. 2021b).

### Constitutive models for stent and balloon

**Fig. 3:**
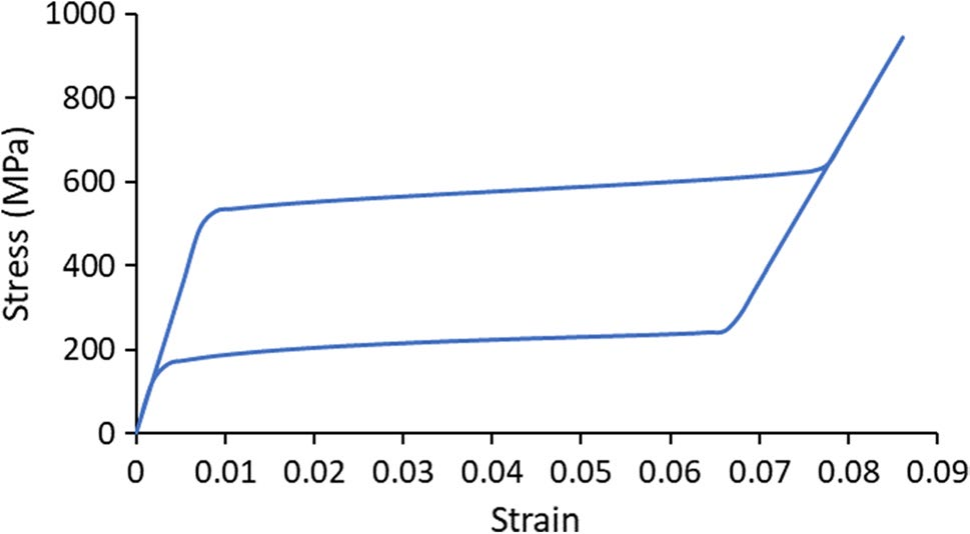
Stress–strain response of nitinol simulated using superelastic model

The self-expandable stent considered in this study was made of superelastic nitinol (nickel titanium). The mathematical equations for the superelastic model are given in Auricchio and Taylor (1997) and Auricchio et al. (1997) with the model parameters given in Azaouzi et al. (2013). The simulated stress–strain response of the nitinol under uniaxial tension is shown in Fig. 3. The non-compliant balloon used for pre-dilation was assumed to be made of polyethylene terephthalate, with density of 1.4E-9 t/mm^3^, the Young’s modulus of 2000 MPa and the Poisson’s ratio of 0.44 (Goodfellow 2018).

## Finite element model

### Models for artery, plaque, balloon, stent and tube

The modelled two-layer femoropopliteal artery had an inner diameter of 4 mm and a length of 50 mm. The diameter of femoropopliteal arteries ranges from 2.5 mm to 11.5 mm (Sandgren et al. 1999; Spector and Lawson 2001; Tayal et al. 2016). The 4 mm diameter used in this study is within this range, although it is a relatively small femoropopliteal artery. For a larger vessel, a larger stent will simply be used and there would be no significant difference in terms of mechanical interaction between stent and vessel of different sizes. Hence, the vessel size is not expected to have significant impact on the model results. The adventitia and media layers had thicknesses of 0.41 mm and 0.74 mm, respectively (Wong et al. 1993). The contribution of the extremely thin intima layer to artery deformation was negligible and, thus, was not considered in this study (He et al. 2020c).

In the middle of the artery, the plaque was modelled as a constant thickness layer, with a length of 18 mm and a stenosis rate of 50% (i.e. an inner diameter of 2 mm). The plaque considered in this work is a highly local lesion which normally has a length between 9.8 and 66 mm (Roberts et al. 2014; Rocha-Singh et al. 2021). The 18 mm length of plaque considered in this study is within this range. In clinical setting, the stent to be implanted should be slightly longer than the plaque; therefore, a Zilver stent of 20 mm in length was used in the work, considering the 18 mm long plaque. A relatively longer stent would be needed for a longer lesion. However, as the design of the Zilver stent was open cell and each crown was relatively independent from each other, the deformation of a longer stent would be very similar to that of a shorter stent. Therefore, the lesion length should not significantly affect the results obtained in this work. Clinically, even moderate stenosis (e.g. 50% considered in this study) may cause problems in feet or legs and, thus, needs treatment. Also, pre-dilation will be performed if the stent cannot be inserted into the diseased artery (including moderate stenosis) or it will be hard for the stent to expand the artery (presence of calcification). Nevertheless, the focus of this study was on a comparative study of stenting outcomes and fatigue performance with and without pre-dilation. As long as a consistent approach is used, the general conclusions regarding the effect of pre-dilation on fatigue performance of nitinol stent should be valid for more severe stenosis (i.e. greater than 50%).

**Fig. 4:**
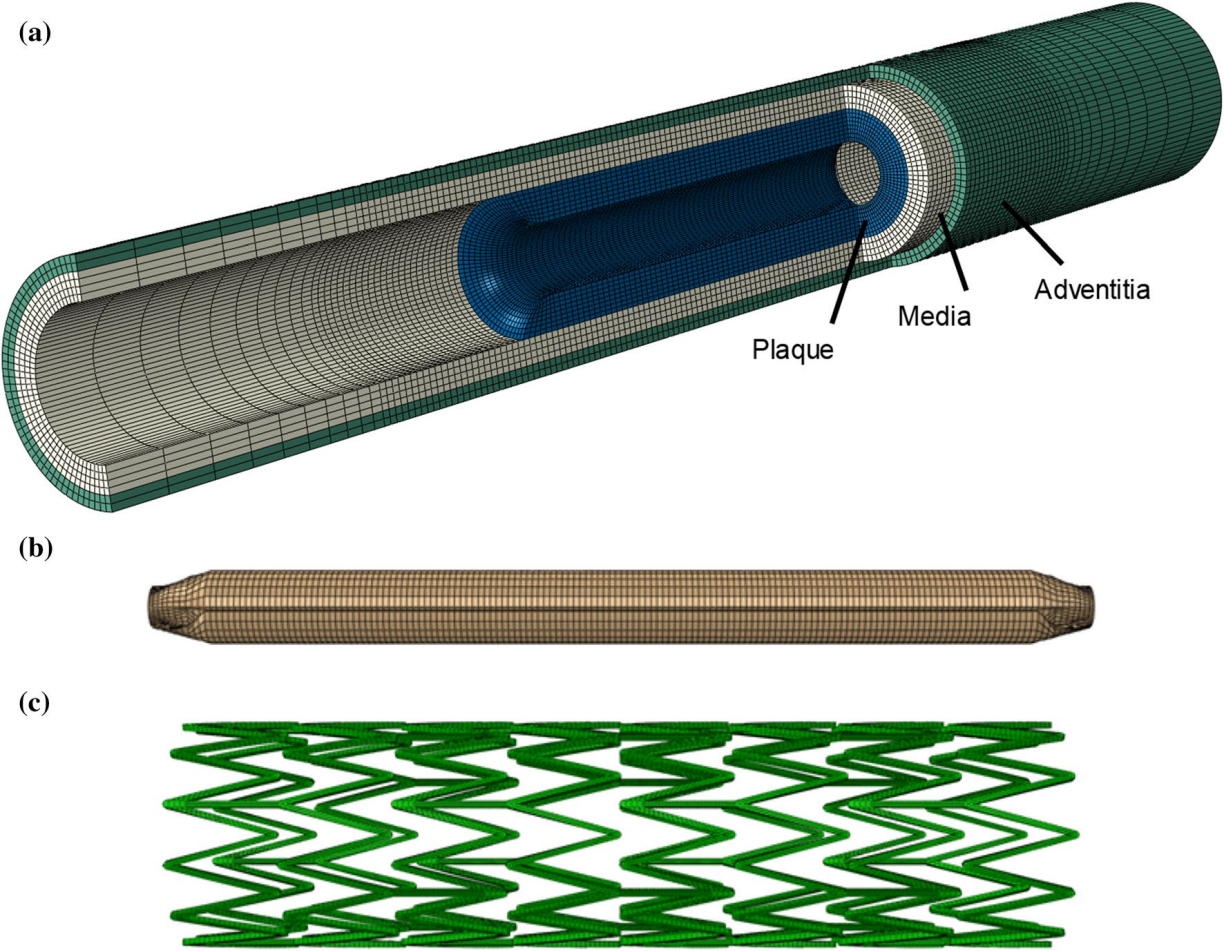
Finite element models: **a** femoropopliteal artery–plaque assembly; **b** pre-dilation balloon; **c** Zilver stent

The artery and the plaque were meshed with hexahedral elements with reduced integration (C3D8R) using Abaqus (Fig. 4a). The adventitia and media layers, and plaque were meshed radially with 2, 4 and 8 rows of elements, respectively. The artery was meshed with a bias control in longitudinal direction, i.e. the element size gradually increasing towards both ends of the artery. A model of a tri-folded balloon was created to simulate pre-dilation, with a length of 21 mm and a nominal diameter of 4 mm (Fig. 4b), meshed with three-dimensional four-node membrane elements with reduced integration (M3D4R). The Zilver Flex® Vascular Self-Expanding Stent (Cook Medical, USA) was modelled using Abaqus CAE, with a length of 20 mm, an outer diameter of 5 mm and a strut thickness of 125 µm (Fig. 4c), meshed with C3D8R elements. A model of a linear elastic tube was built for crimping and releasing the Zilver stent in the diseased artery.

### Boundary conditions

All simulations were implemented with the Abaqus (2017) explicit solver; each employed 160 cores on a high-performance computer cluster and took about two months to finish. Pre-dilation was simulated by applying a peak inflating pressure of 1.8 MPa to the inner surface of the balloon. A general hard contact with a coefficient of friction of 0.2 was used to model the interaction between the balloon and the artery (Dordoni et al. 2014). Then, the stent was gradually crimped to an outer diameter of 2 mm by applying an inward radial displacement on the tube around it. The same properties used for the balloon–artery interaction were used to model the interaction between the stent and the tube. Subsequently, a longitudinal displacement was applied to the tube to gradually remove it and release the stent in the diseased artery. The interaction of the stent with the artery was added in this step, using the same properties as those employed for the balloon–artery interaction. The displacements of both ends of the artery were fixed throughout the simulations to consider the constraints imposed by the rest of the artery.

### Loading conditions

Loadings of blood pressure, bending, torsion, axial compression and their combination were applied to the artery to study the fatigue behaviour of the implanted nitinol stent. The simulations for the blood pressure and the rest loading conditions ran for 20 and 5 loading cycles, respectively. In this step, the interaction between the stent and the artery remained while the tube was removed. A realistic waveform with fluctuations was used to model the blood pressure, with a diastolic value of 80 mmHg (0.01 MPa) and a systolic value of 120 mmHg (0.015 MPa; Qiu et al. 2018).

**Fig. 5:**
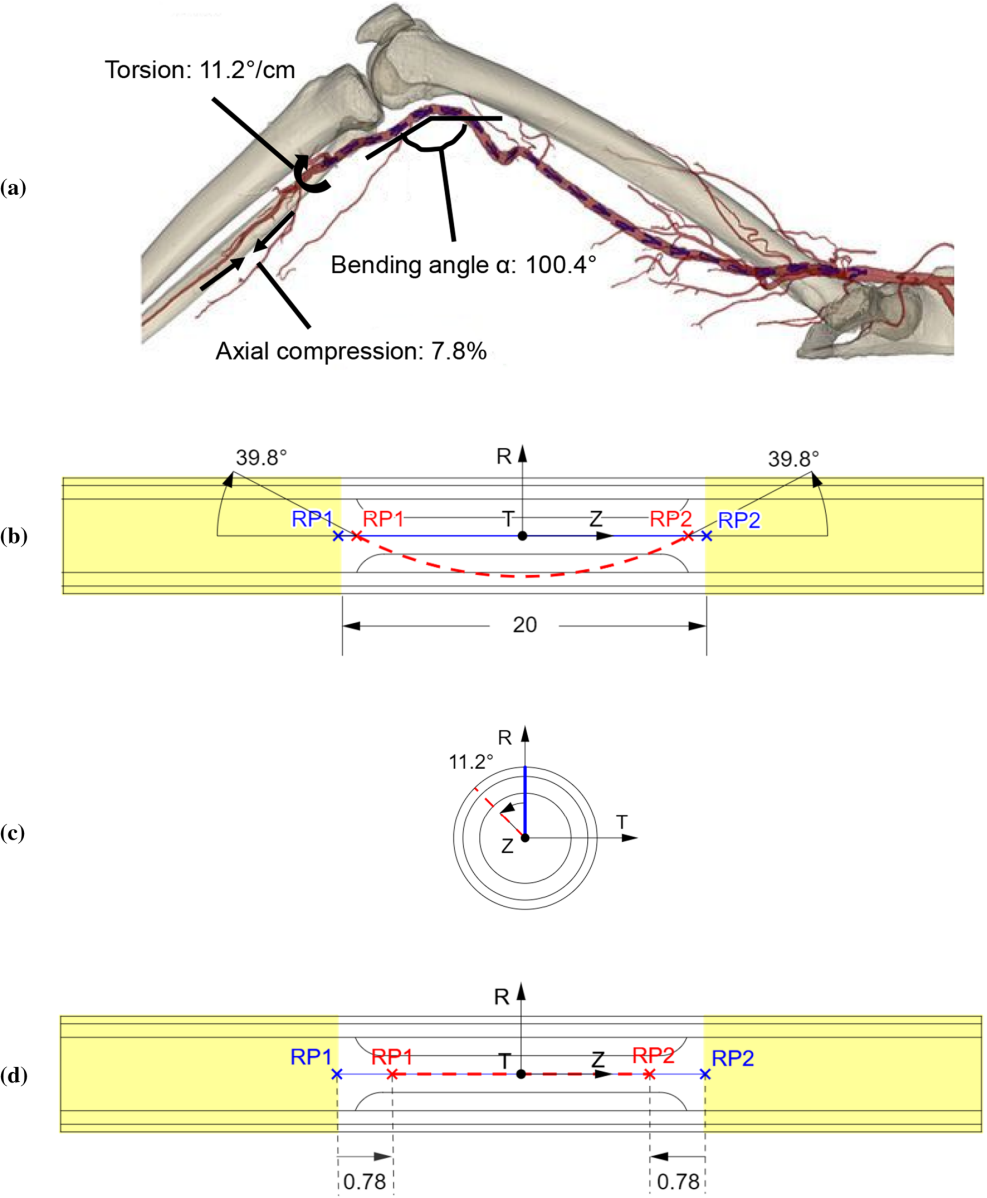
**a** Computed tomography of limb’s flexion state demonstrating walking posture (Desyatova et al. 2017), with most severe deformations in stented femoropopliteal artery during walking (not drawn to the exact positions). Schematics of **b** bending, **c** torsion and **d** axial compression (constrained regions: yellow; initial condition: blue; deformed condition: red). All dimensions of length in mm (not to scale)

In Desyatova et al. (2017), Poulson et al. (2018) and MacTaggart et al. (2019), the peak deformations for bending, torsion and axial compression were measured by comparing marker positions for the walking ending angle (110°) and standing (180°) postures. These papers reported deformations for the superficial femoral artery, the adductor hiatus segment and the popliteal artery, and the popliteal arteries experienced the largest deformation. The exact locations with the most severe deformations in the popliteal arteries were not indicated in the papers, but it is most likely to happen in the section behind the knee during daily walk. The most severe deformations measured for the popliteal arteries were taken as the worst case for walking-induced motion on fatigue failure of implanted nitinol stents (Fig. 5a). The mean values of torsion were taken from Desyatova et al. (2017), while axial compression and bending from Poulson et al. (2018) measured for the popliteal artery in the walking posture, plus the corresponding increases or restrictions due to the presence of the Zilver stent given in MacTaggart et al. (2019). The schematics for loadings of bending, torsion and axial compression are shown in Fig. 5b–d, respectively, where the initial and deformed (peak load) conditions are in blue and red colours, respectively. Based on kinematic coupling, all degrees of freedom, except the radial displacement, of the nodes in the non-stented regions (yellow in Fig. 5) were constrained to the two reference points—RP1 and RP2, respectively. Specifically, rotations of ∓ 39.8° and ∓ 11.2° around the *T*- and *Z*-axis and displacements of ± 0.78 mm along the *Z*-axis were applied to RP1 and RP2 simultaneously to simulate bending, torsion and axial compression loadings, respectively. All cyclic loadings had a sine waveform with a frequency of 1 Hz (Ji and Pachi 2005). Constraints of the artery ends were removed for these steps.

### Fatigue assessment

*FDA recommendations for nitinol stent fatigue characterization* (Cavanaugh et al. 2006) states: “FE analysis model outputs can take many forms: equivalent strains/stresses, principal strains/stresses, or von Mises stresses at specific locations on the stent are commonly reported. Once the model is run and results extracted, safety factors can be calculated and presented both as numerical values in tabular format and in comparison to a constant-life curve formulated from an appropriate stress-based or strain-based relationship.” Also, the fatigue life of nitinol stents is designed for 10^7^ cycles (i.e. 10 years), which belongs to high cycle fatigue regime and can be assessed using stress-based approach. Therefore, stress was used for nitinol fatigue analysis in this study. Specifically, the Goodman’s rule was employed, expressed as

8 $$\begin{array}{c}\frac{{\sigma }_{a}}{{\sigma }_{\mathrm{fat}}}+\frac{{\sigma }_{m}}{{\sigma }_{\mathrm{ts}}}=1,\end{array}$$

where ${\sigma }_{a}$ is the stress amplitude, ${\sigma }_{m}$ is the mean stress, and ${\sigma }_{\mathrm{fat}}$ and ${\sigma }_{\mathrm{ts}}$ are the fatigue stress limit and the ultimate tensile strength of the nitinol, respectively. In this study, ${\sigma }_{\mathrm{fat}}$ and ${\sigma }_{\mathrm{ts}}$ were taken as 272 MPa and 878 MPa, respectively (Pelton et al. 2008; Lin et al. 2009). A Python script was written to extract the peak and valley stresses (von Mises stress) for all the elements in the modelled stent in the last cycle for each loading case. They were used to calculate the mean stress and the stress amplitude and then plotted in the Goodman’s diagram for fatigue assessment.

## Results

### Deformation during pre-dilation

**Fig. 6:**
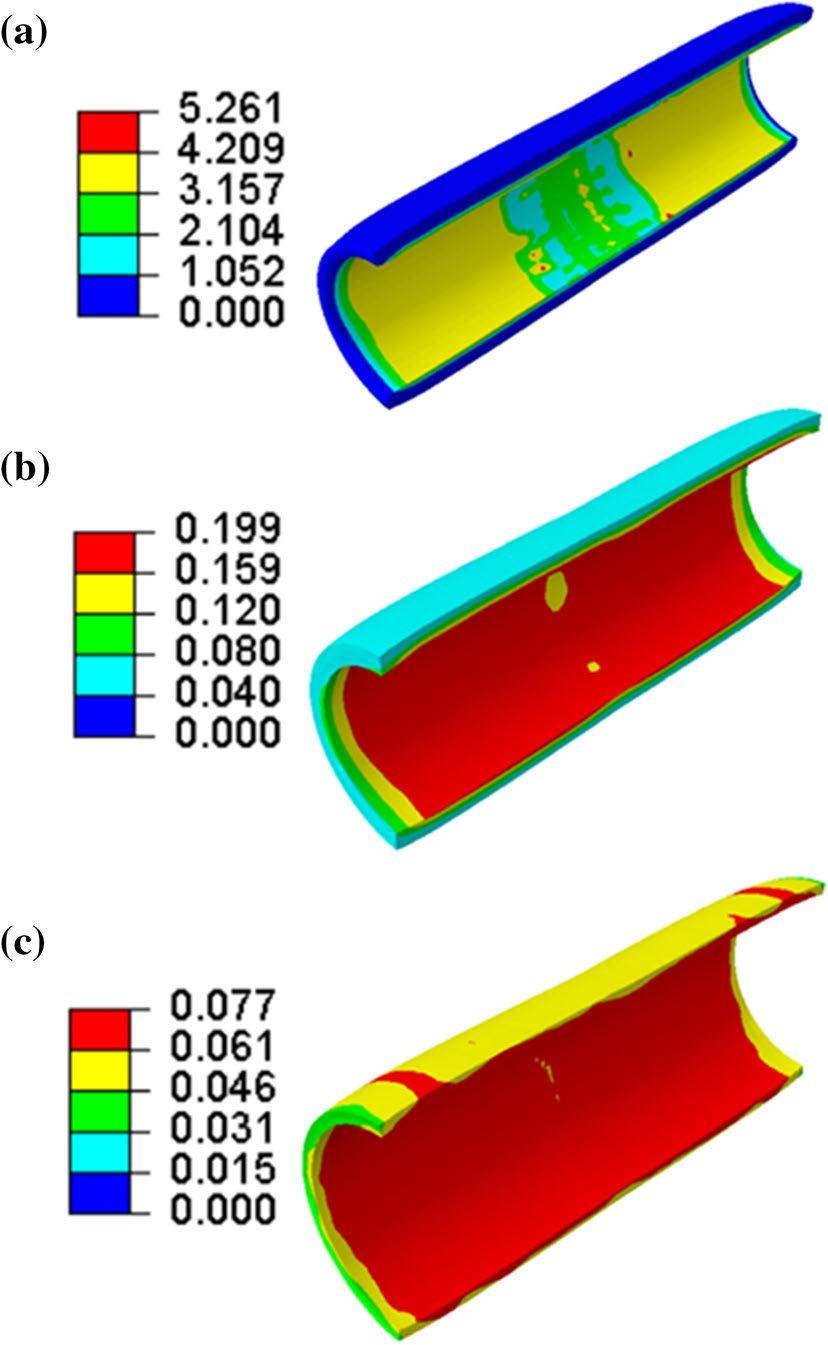
Contour plots of von Mises stress (in MPa) in **a** plaque, **b** media and **c** adventitia (diseased region) at peak inflating pressure of pre-dilation

**Fig. 7:**
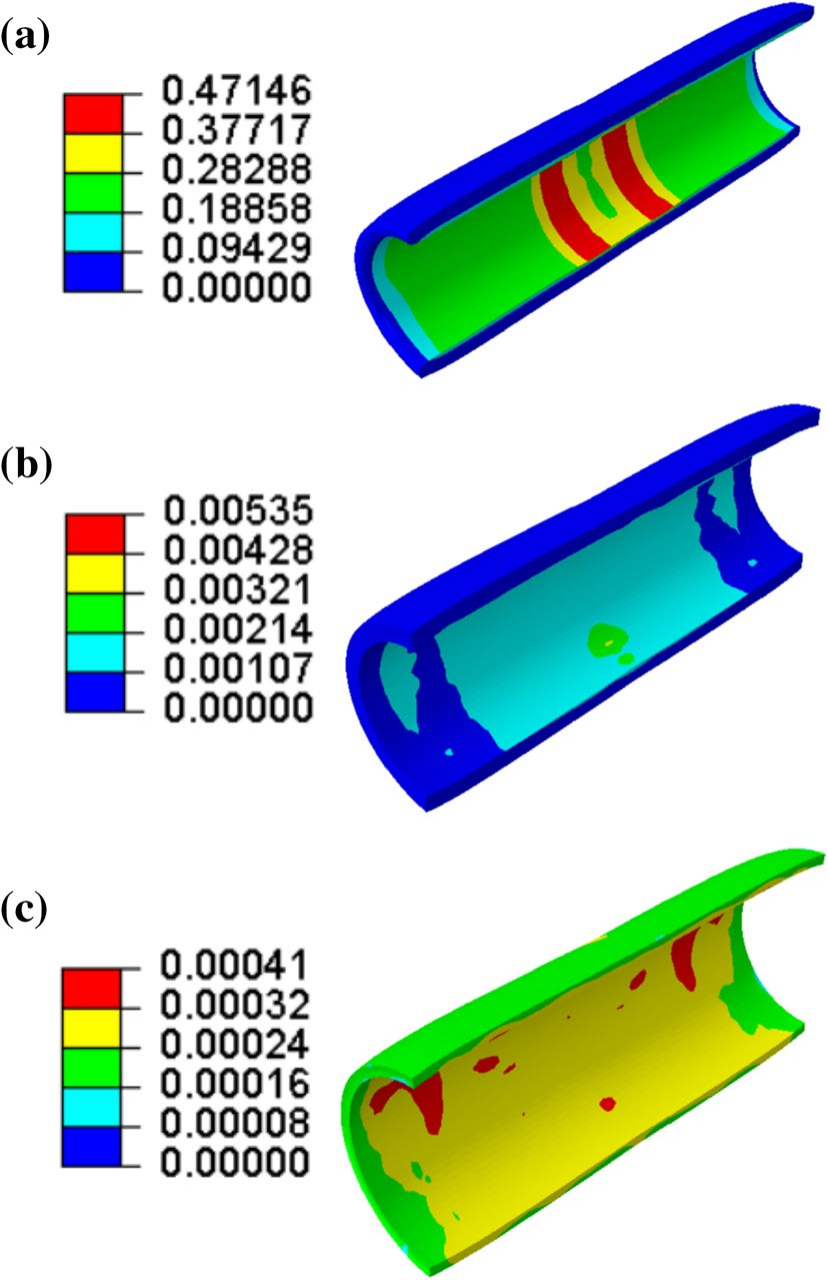
Contour plots of dissipation energy (in mJ/mm^3^) in **a** plaque, **b** media and **c** adventitia (diseased region) at peak inflating pressure of pre-dilation

Numerical simulations with the developed FE models were used to assess various processes related to the use of pre-dilation. Figures 6 and 7 show the contour plots of von Mises stress and dissipation energy in the plaque, media layer and adventitia layer (diseased region) at the peak inflating pressure of pre-dilation. The dissipation energy was used to quantify extent of the damage in the tissue. The von Mises stress in the middle parts along the length of each layer was lower than in the nearby regions; however, the damage was mainly concentrated in these parts, especially, in the plaque and the media layer. The dissipation energy is related to the maximum strain energy in the deformation history, while the stress is calculated from the derivative of strain energy with respect to the deformation gradient. Hence, the dissipation energy and the stress may be not in a positive relationship, i.e. regions with higher level of stress may not necessarily had larger dissipation energy. This resulted in different distributions of the von Mises stress and damage in the plaque and arterial layers, as shown in Figs. 6 and 7. The levels of both von Mises stress and damage decreased through the thickness—from the inner surface to the outer one of the plaque and arterial layers; with the highest magnitudes in the plaque, followed by those in the media and adventitia layers.

### Deformation after stent deployment

**Fig. 8:**
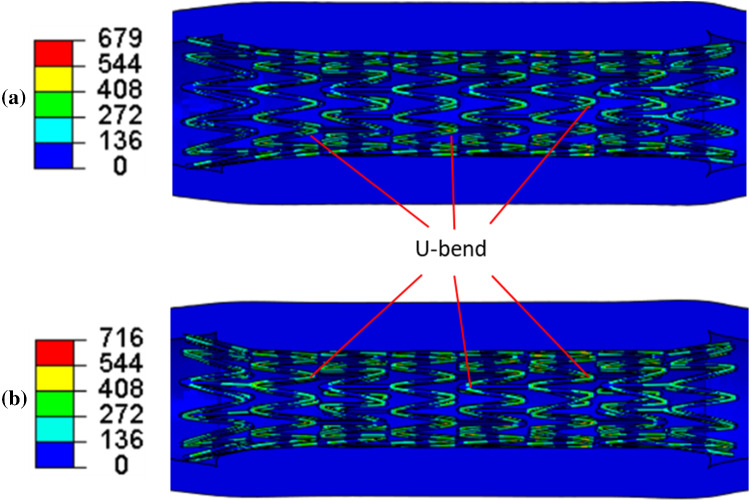
Contour plots of von Mises stress (in MPa) in stent after deployment **a** with and **b** without pre-dilation, where U-bends are also indicated

**Fig. 9:**
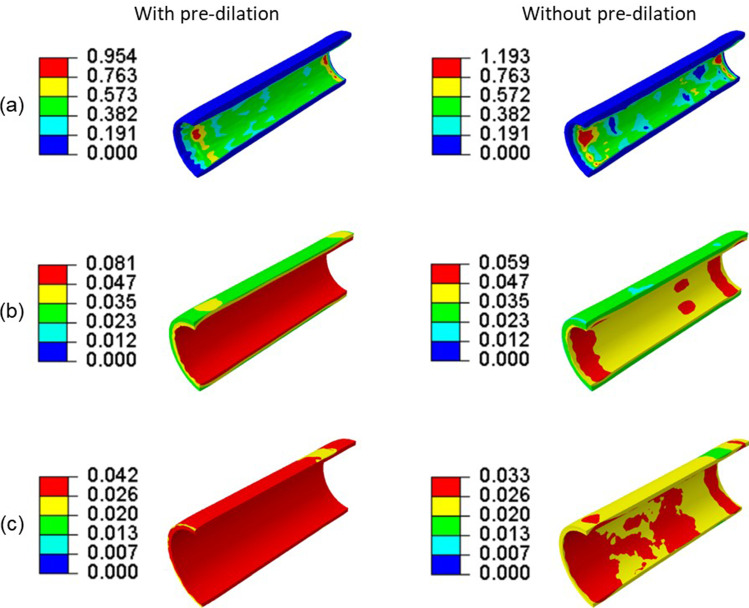
Contour plots of von Mises stress (in MPa) in **a** plaque, **b** media and **c** adventitia (diseased region) after stenting with pre-dilation and without it

Considering the multiaxial stress state in the stent, the von Mises stress after deployment with pre-dilation and without it is plotted in Fig. 8. Apparently, the high-stress regions were dominantly located at the U-bend parts in both cases. Here, the U-bend parts refer to the “U”-shaped struts in each crown of the stent, as illustrated in Fig. 8. The maximum von Mises stress in the stent without pre-dilation was much higher than that with pre-dilation (716 MPa vs. 679 MPa). Both stents showed a dog-boning effect. Apparently, more severe stenosis will limit the expansion of stent during deployment process. The stenosis modelled in this study was more severe in the middle and less severe towards the ends of the plaque; therefore, the stent expanded less in the middle and more towards the ends, leading to a dog-bone shape. This was also observed in clinical cases (Hernández-Enríquez et al. 2018; Włodarczak et al. 2022). In addition, the von Mises stresses developed in the diseased arteries after stenting with pre-dilation and without it are presented in Fig. 9. The high levels of von Mises stress were located on the inner surface of the plaque due to its direct contact with the stent. Although the maximum stress in the plaque with pre-dilation was lower than that without pre-dilation (0.954 MPa vs. 1.193 MPa), the overall stress in the plaque with pre-dilation was higher than that without pre-dilation; this was also the case for the media and adventitia layers. This can be explained by a larger lumen in stenting with pre-dilation, i.e. higher arterial deformation than that without pre-dilation (3.76 mm vs. 3.46 mm for the average lumen diameter).

### Fatigue performance

**Fig. 10:**
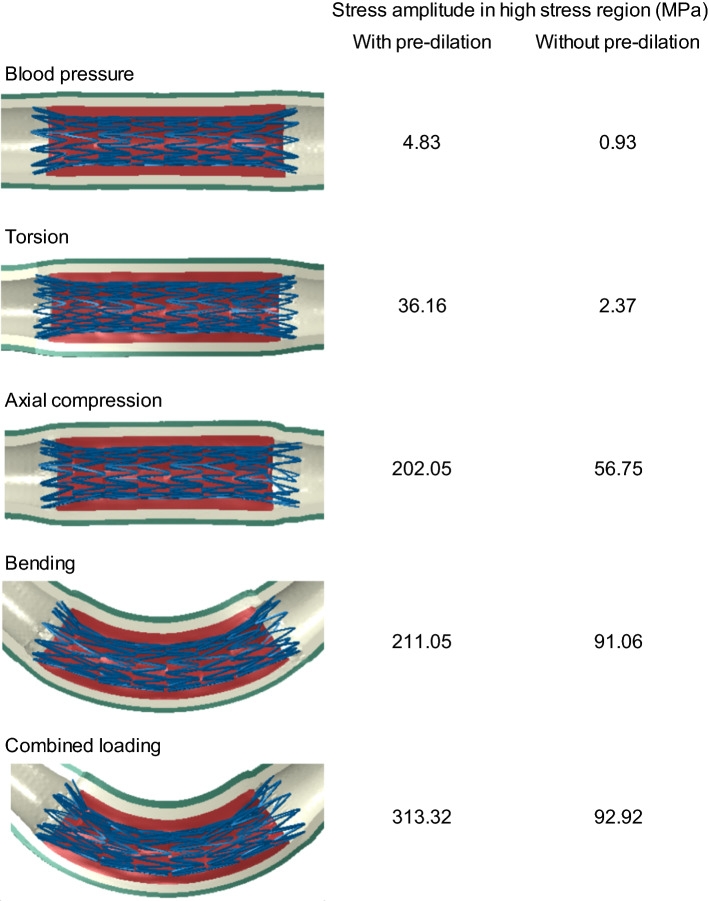
Deformations of stent and artery at peak loading events for five cases of loading, with respective stress amplitude in high-stress region of stents indicated for cases with pre-dilation and without it

**Fig. 11:**
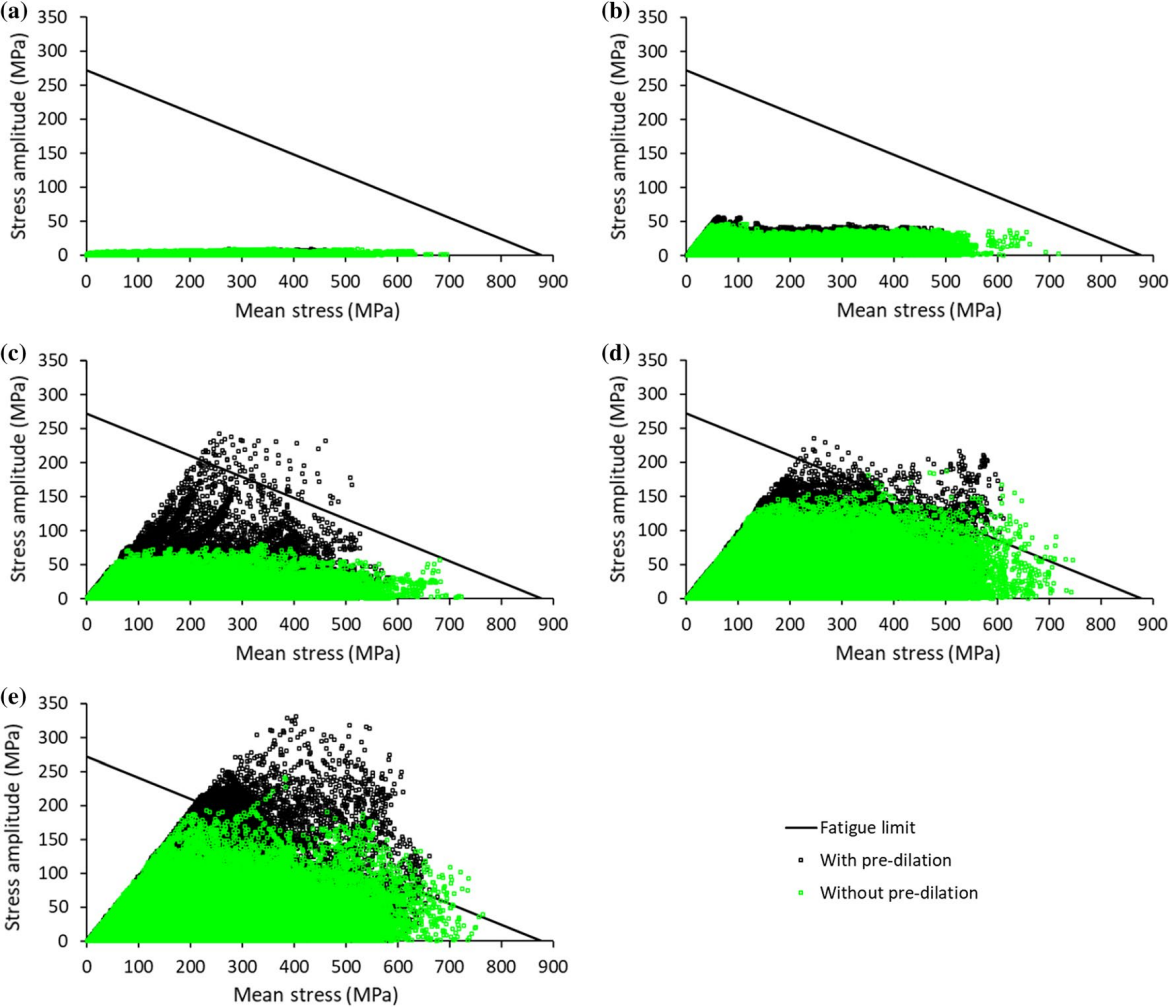
Goodman’s diagrams for fatigue performances of stents with pre-dilation and without it for five studied loading cases: **a** blood pressure; **b** torsion; **c** axial compression; **d** bending; **e** combined loading

Deformations of the stent and the artery at peak loading condition under five types of loading—blood pressure, torsion, axial compression, bending and their combination—are plotted in Fig. 10, with the stress amplitude in the high-stress region of the stent indicated for cases with pre-dilation and without it. The stress amplitude in this region increased from one loading case to another, while the amplitudes with pre-dilation were always higher than those without it under all fatigue loading conditions. The fatigue performance of stent with pre-dilation and without it are compared in Fig. 11 for all these five loading cases. Apparently, the stress amplitude in the stent with pre-dilation was always higher than that without it, while the mean stress, on the contrary, was always lower for the former case. However, analysis of the dangerous zone (i.e. above the fatigue limit) for bending, axial compression and combined loadings demonstrated that more elements were in this zone and also further beyond the fatigue limit line for stent deployment with pre-dilation due to the increased levels of the stress amplitude and mean stress in the stent (for instance, an amplitude up to 331.48 MPa and a mean stress up to 675.96 MPa for the case with pre-dilation, compared to an amplitude up to 241.51 MPa and a mean stress up to 763.75 MPa for the case without pre-dilation, in the combined loading case), indicating an increased risk of fatigue failure for the stent.

The effect of different loading types on fatigue failure of the stent were similar for both cases. All the elements were in the safe zone for the pulsatile blood pressure, as a result of low levels of stress amplitude and mean stress. Torsion introduced slightly higher stress amplitudes and mean stresses in the stent compared to the blood pressure, but all the elements were still well within the safe zone. Although there were further increases in stress amplitude and mean stress for axial compression, the situation was somewhat different for the two cases. Some elements were located in the dangerous zone for deployment with pre-dilation, while all the elements were still within the safe zone for the case without it. For bending fatigue, elements were found in the danger zone for both cases, and an increased number of elements were found in the fatigue failure zone for deployment with pre-dilation. The combined loading had the most elements located in the dangerous zone, indicating the highest risk of fatigue failure among all loading scenarios. Also, pre-dilation resulted in an increased risk of stent fatigue failure, consistent with the cases of axial compression and bending fatigue.

### Stress vs. strain approaches

**Fig. 12:**
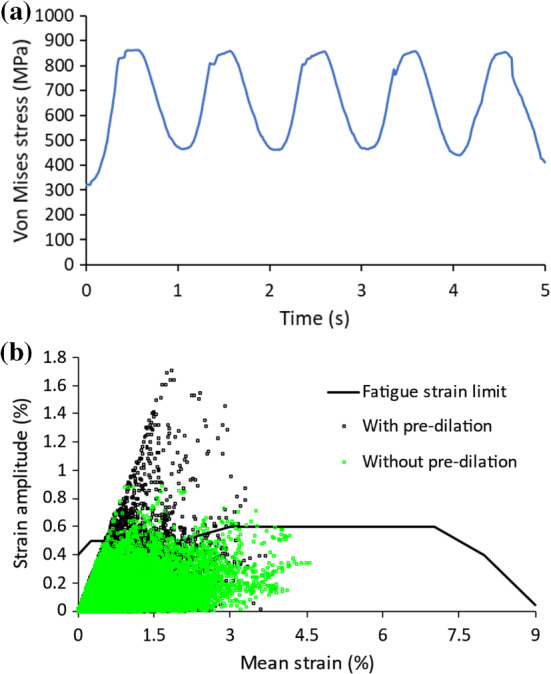
**a** Evolution of von Mises stress in high-stress region in stent under combined fatigue loading; **b** constant life diagram for fatigue performances of stents with pre-dilation and without it for combined loading

Phase change is directly associated with the deformation of the stent during the crimping and releasing. Different locations of the stent could experience different deformation stages, i.e. some in the elastic deformation regime of the two phases (austenite and martensite) and some in the superelastic deformation regime with the involvement of phase transformation. During phase transformation, stress tends to exhibit a plateau behaviour (see Fig. 3). To investigate whether or not phase transformation occurred during the fatigue loading, the stress evolution in the high-stress region of the stent deployed with pre-dilation was extracted and is plotted in Fig. 12a for the combined fatigue loading case (worst case scenario). Apparently, phase transformation did not occur during the combined fatigue loading, as no stress plateau was observed. Plateau mainly occurred during stent crimping and releasing processes, which was associated with phase transformation. In the subsequent fatigue loading regime, the deformation appeared to be dominated by linear elastic behaviour of the alloy (i.e. without phase transformation). Thus, the stress-based approach should be appropriate. Nevertheless, the effective strain amplitude and the mean strain were extracted for the combined loading case and used to assess the fatigue performance of the stent. The constant life diagram using the strain life approach is shown in Fig. 12b, where the fatigue strain limit curve was provided in Pelton (2011). From Fig. 12b (compared to Fig. 11e), the same conclusion can be drawn, i.e. more elements were in the dangerous zone and also further away from the fatigue limit line for stent deployment with pre-dilation, indicating an increased risk of fatigue failure for the stent.

### Tissue damage in media layer

**Fig. 13:**
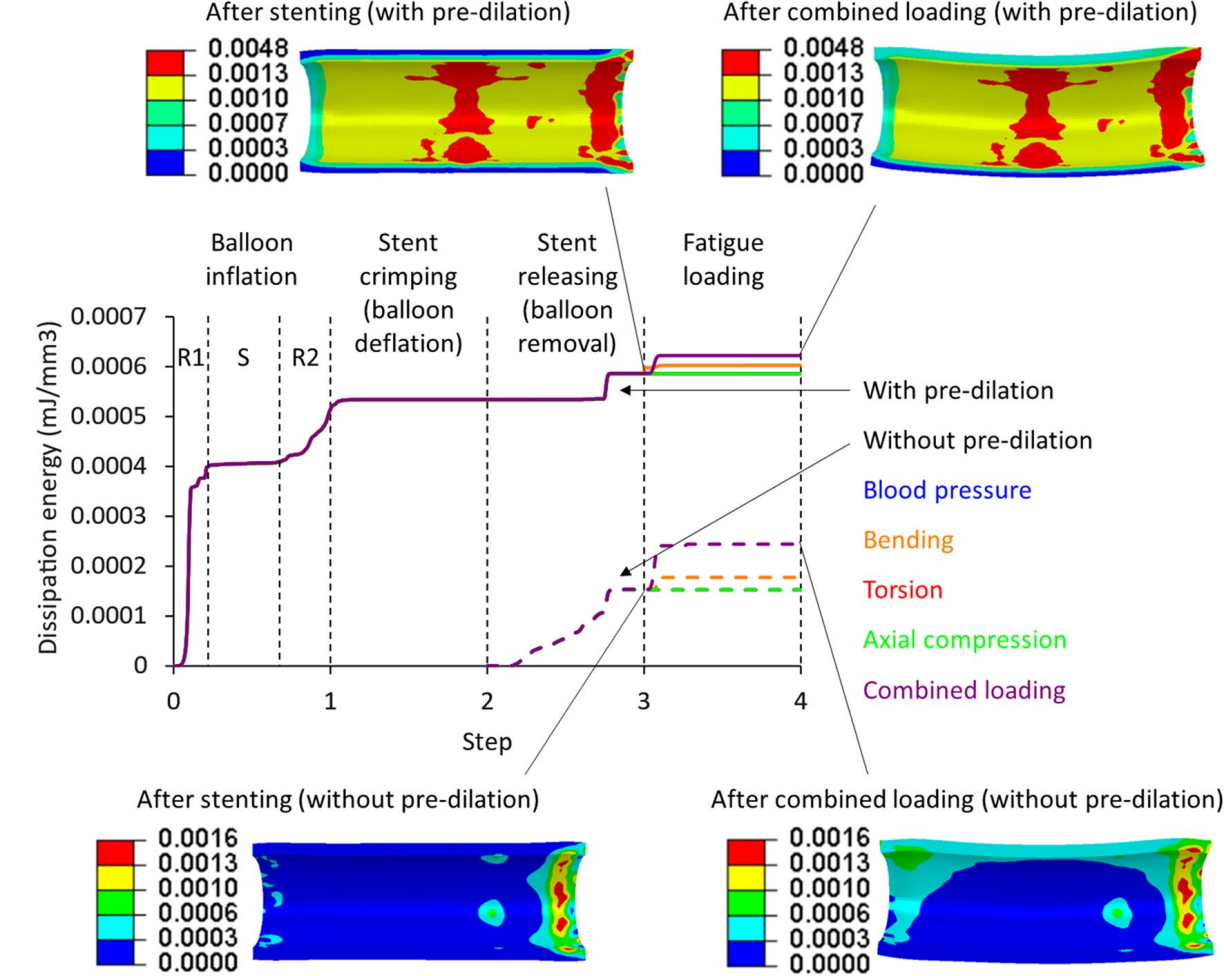
Evolution of average dissipation energy (in mJ/mm^3^) in media layer (diseased region) during pre-dilation, stenting and five studied cases of fatigue loadings, with corresponding contour plots after stenting and combined loading for cases with pre-dilation and without it

Since the neointima formation, the major contribution to ISR, is associated with the stenting-caused tissue damage in the media layer (Farb et al. 2002), the damage accumulated in this layer (diseased region) was assessed and compared for stent deployment with pre-dilation and without it (Fig. 13). The damage accumulation (in terms of dissipation energy) in the media layer during the balloon inflation in pre-dilation experienced stages of rapid (R1), slow (S) and rapid (R2) increases, corresponding to the initial rapid balloon unfolding, slow unfolding and slight expansion of the balloon. Both stenting with pre-dilation and without it introduced (further) damage in the media layer, but much less compared to pre-dilation. For both cases, the damage in the media layer did not increase any further in three cases of loading (blood pressure, torsion and axial compression), but grew under the bending and combined fatigue loadings in their first cycle. The contour plots of dissipation energy are also presented in Fig. 13 for the media layer (diseased region) after stenting and combined loadings with pre-dilation and without it. Apart from the right end of the media layer for both cases, the high damage was located in the middle for the case with pre-dilation. The asymmetric distribution of the damage was caused by the stent release from the left to the right. Its early released left crowns facilitated the expansion of right crowns, resulting in larger deformation in the right end of the artery and, subsequently, higher local damage in the media layer. However, the direction of stent unsheathing caused only slight variability in the tissue damage as shown in Fig. 13 (ranging from 0 to 0.0048 mJ/mm^3^), which was not significant.

### Evaluation of in-stent restenosis

**Fig. 14:**
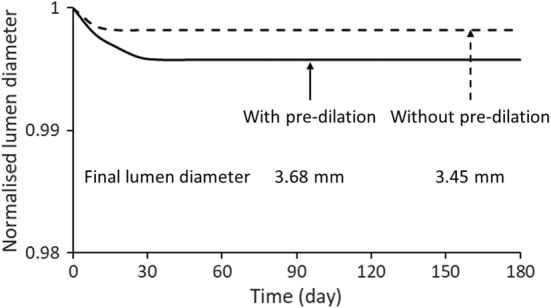
Predicted evolution of normalised lumen diameter over 6 months for cases with pre-dilation and without it for combined loading

The predicted evolutions of ISR for the most severe conditions of combined loading with pre-dilation and without it using the correlation of ISR with the dissipation energy and time (see Appendix) are plotted in Fig. 14. By substituting the level of final average dissipation energy in the media layer (diseased region) into the correlation, the lumen diameter, normalised by its initial post-stenting value, was predicted for 6 months of tissue growth (Fig. 14). Apparently, although the development of ISR with pre-dilation was more than that without it, both cases were not severe.

It should be noted that this study did not simulate the actual process of ISR or vessel remodelling, but predicted the ISR based on a static correlation constructed in our previous study (He et al. 2020c; see Appendix). Specifically, in our previous study (He et al. 2020c), we simulated the development of ISR after stenting by removing the dynamic interaction between the stent and the vessel, i.e. the dynamic stress reallocation was ignored in the study. This was due to the limitation of the growth model, which was unable to simulate the dynamic growth of tissue over the stent.

## Discussions

This study is the first attempt to investigate with advanced FE simulations the effects of pre-dilation on the deployment of nitinol stents in terms of lumen gain and tissue damage. Currently, the self-expandable nitinol stent is the most advanced device to treat the diseased femoropopliteal artery thanks to its superelastic behaviour. However, it usually requires performing a pre-dilation to enable the insertion of the stent into the diseased artery as well as facilitation of its expansion. Thus, it is important to understand its potential benefits for the outcomes of stent treatment. Our results showed that pre-dilation facilitated the stent to expand the diseased artery by softening the vessel wall in advance, thus resulting in a further lumen gain as well as lower stresses in the stent after deployment. However, it also resulted in a higher level of tissue damage in the media layer, in agreement with the results in He et al. (2020a).

Furthermore, this study also investigated the impact of pre-dilation on the fatigue performance of the nitinol stent implanted in the femoropopliteal artery for the first time. He et al. (2021a, b) demonstrated that for stenting with pre-dilation, the pulsatile blood pressure did not contribute significantly to the fatigue failure of the stent and the combined loadings imposed the highest risk in this regard, with main contribution from bending followed by axial compression and torsion. This study confirmed that it was also the case for stenting without pre-dilation. However, under all fatigue loading regimes, the stent deployed without pre-dilation had a higher mean stress, while the one deployed with pre-dilation had a larger stress amplitude. This was because pre-dilation facilitated the expansion of the stent in the artery, thus resulting in decreased deformation and a reduced stress level in the stent after deployment. The stent was also easy to deform when the artery was subsequently subjected to external fatigue loading, leading to increased stress amplitudes in the stent. Although the stent deployed after pre-dilation had a reduced mean stress, the high stress amplitude increased the risk of fatigue failure of the stent. Preparing the artery with pre-dilation is necessary when treating occlusive disease. On the other hand, the bending and combined loading regimes increased the damage level in the media layer in their first cycle. However, as the amount of the increased damage was not significant and its level barely increased in the following cycles, these repetitive biomechanical loadings should not be a critical problem in terms of causing the tissue damage in the media layer.

The neointimal growth, the major cause of ISR, was significantly associated with the stenting-induced media injury (Hoffmann and Mintz 2000; Farb et al. 2002). Such injury stimulated the smooth muscle cells to proliferate and migrate from the media layer to the intima layer to form neointimal hyperplasia. Our predicted development of ISR based on the stenting-induced damage in the media layer with pre-dilation was more severe than that without it, in agreement with the animal results (Harnek et al. 2002). The study in Harnek et al. (2002) reported results from healthy pigs with no vascular disease, and therefore, it ignored the luminal gain from pre-dilation in the first place. This beneficial fact cannot be ignored in occlusive disease where the pre-dilation treatment is necessary in order to widen the lumen. The aim of pre-dilation is to facilitate the stent to achieve larger lumen gain. However, larger lumen gain will result in further damage in the arterial wall and, subsequently, higher risk of ISR. They are two sides of a coin and seem inevitable. Furthermore, in both cases, the predicted ISR was 8% and 13.75% for stenting with and without pre-dilation, respectively, which was believed to be rather small. Hence, the predicted risk of ISR caused by pre-dilation, implantation of nitinol stent and physiological and biomechanical loadings was quite low, confirming the clinical observations (Schillinger et al. 2006; Fusaro et al. 2013). The factors associated with a high risk of ISR include long and heavily calcified lesion, diabetes mellitus and stent fracture, which were not considered in this study and can be investigated in future work.

There are some limitations in this study. Firstly, residual stresses in the vessel wall are likely to induce a higher level of stresses in the stent and artery, leading to a higher risk of fatigue failure of the stent. However, residual stress effect was not considered in this study and neither in other literature. This is because the Abaqus/Explicit solver, used to simulate the stenting procedure, is unable incorporate the residual stresses of the vessel wall into the simulations due to the consideration of material compressibility (i.e. the material could not be set as incompressible in Abaqus/Explicit). In another word, the residual stress solutions obtained analytically for the artery are only valid for an incompressible hyperelastic material (Holzapfel et al. 2007; Holzapfel and Ogden 2010). Therefore, future work is required to resolve this issue in order to investigate the effect of residual stress in the vessel wall on the mechanical deformation and fatigue life of nitinol stents.

Secondly, although we did not consider calcified plaque or plaque rupture in this study, the reported effect of pre-dilation on stent performance and fatigue failure is expected to be still valid. This is because pre-dilation facilitates the expansion of the stent in the diseased artery, irrespective the plaque type, resulting in lower stress/strain level in the stent after deployment. The stent is then easier to deform when the artery is subsequently subjected to external fatigue loading, leading to increased stress amplitudes in the stent and a higher risk of fatigue failure. Also, the plaque model was idealised as a single, uniform and symmetric layer, while the plaque normally has a complex morphology and is often not concentric, with varied mechanical properties. Therefore, the contact between the plaque/artery and stent may be very different from that in the current model, which may increase local strain concentration and shorten the fatigue life of the deployed nitinol stent. Hence, patient-specific models are recommended in future studies, based on high-resolution clinical imaging of diseased arteries in patients.

Additionally, it should be noted that post-dilation is often performed after the deployment of nitinol stents, causing additional deformation and further increase of stress/damage in the stent and artery. Hence, it could increase the risks of stent fracture and ISR. In addition, the effects of the length of pre-dilation and the potential for vessel recoil on the performance of nitinol stents may also affect the fatigue performance of nitinol stent, which needs to be studied in future work. Also, this study only considered the ISR triggered by damage. However, it has been demonstrated the role of hydrodynamic forces in tissue growth, in particular in the long term (Chen et al. 2022; Zhang et al. 2022). In this regard, a more accurate prediction of ISR will need to consider CFD simulation of the stented artery in addition to accounting for tissue damage in future work. It should be also noted that clinical data are not available at the moment to further support these conclusions, but can be accomplished through in vitro and/or in vivo studies in future work.

## Conclusions

The effect of pre-dilation on performance of the nitinol stent implanted in the femoropopliteal artery was investigated. Simulations based on the developed FE models were carried out for deployment of the nitinol stent with pre-dilation and without it, followed by repetitive physiological and biomechanical loadings. Pre-dilation resulted in lower stresses in the stent but a higher level of damage in the media layer after stenting, compared to the case without pre-dilation. For both cases, the fatigue failure of the stent could hardly be caused by the pulsatile blood pressure, while the combined loading induced the highest risk, with the most contribution from bending, followed by axial compression and torsion. Still, the risk of fatigue failure for stent deployed with pre-dilation was higher than that without it. The pre-dilation, implantation of the nitinol stent and follow-on fatigue loadings did not cause severe tissue damage and growth.

## Figures and Tables

**Fig. 15:**
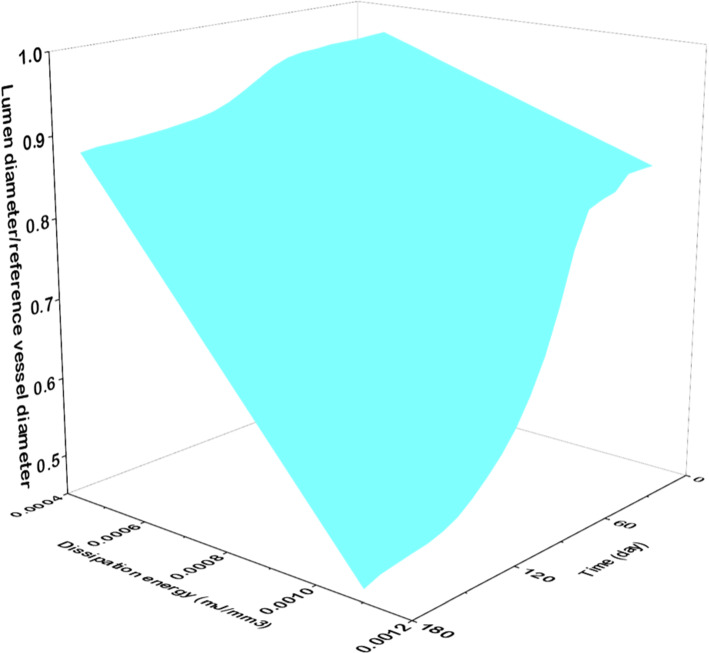
Correlation of in-stent restenosis with dissipation energy and time

## Appendix

The growth model used for simulating the ISR was adopted from Fereidoonnezhad et al. (2017), based on a consideration of an intermediate configuration between the reference and the current ones. Since the neointima formation is associated with the inflammatory reaction to the stenting-caused damage in the media layer (Farb et al. 2002), the growth model was only applied to this layer in the simulations. The deformation gradient **F** is multiplicatively decomposed into an elastic part ${\mathbf{F}}_{e}$ and a growth part ${\mathbf{F}}_{g}$:

A1 $$\begin{array}{c}\mathbf{F}={\mathbf{F}}_{e}{\mathbf{F}}_{g}.\end{array}$$

Substituting Eq. (A1) into ${\varvec{\upsigma}}=\frac{1}{J}\frac{\partial \psi }{\partial \mathbf{F}}\cdot {\mathbf{F}}^{\mathrm{T}}$, we have

A2 $$\begin{array}{c}\varvec{\sigma} ={\mathbf{F}}_{g}^{-1}{{\varvec{\sigma}}}_{e}{\mathbf{F}}_{g}^{-T}.\end{array}$$

A specific form is required for the growth tensor ${\mathbf{F}}_{g}$ in order to fulfil the constitutive formulation. The growth of the anisotropic arterial tissue is expected to be anisotropic; however, as no experimental data are available to characterise the anisotropic neointimal growth, an isotropic growth tensor was adopted here and expressed as:

A3 $$\begin{array}{c}{\mathbf{F}}_{g}={\lambda }_{g}\mathbf{I},\end{array}$$

where ${\lambda }_{g}$ is the growth stretch.

Substituting Eq. (A3) into Eq. (A2) leads to

A4 $$\begin{array}{c}\varvec{\sigma} ={{\varvec{\sigma}}}_{e}/{\lambda }_{g}^{2}.\end{array}$$

To associate with the damage parameter ${D}_{g}$ in the modified HGO-C damage model, the growth stretch is proposed as (similar to the work of Fereidoonnezhad et al. (2017))

A5 $$\begin{array}{c}{\lambda }_{g}=\prod_{\alpha =1}^{N}\sqrt{{\lambda }_{g,\alpha }}=\prod_{\alpha =1}^{N}\mathrm{exp}\left\{\frac{k}{6{\beta }^{2}}\left[1-\left(1+\beta t\right){e}^{-\beta t}\right]\langle {D}_{g,\alpha }-{D}^{th}\rangle \right\},\end{array}$$

with

A6 $$\begin{array}{c}{D}_{g,\alpha }=\frac{1}{{r}_{f}}\mathrm{erf}\left(\frac{{\overline{\psi }}_{f,\alpha }^{\mathrm{max}}}{{m}_{f}}\right).\end{array}$$

where $k$ and $\beta$ are the material parameters, and *D*^*th*^ is the threshold value for the growth to start.

A VUMAT subroutine was coded to realise the growth model in Abaqus/explicit computationally (He et al. 2020b). In the tissue growth step (6 months), the media began to grow based on the damage caused by the stent deployment (i.e. the development of ISR).

Evaluation of ISR was based on a direct correlation of lumen narrowing with the tissue damage over the time after stent implantation obtained in He et al. (2020c). Specifically, using the FE approach, the development of ISR after stenting was simulated based on a tissue growth model, linking with tissue damage. The level of stenting-induced tissue damage in the media layer (diseased region) was represented by the average dissipation energy. The correlation of the lumen diameter with respect to the reference vessel diameter, dissipation energy and growth time is given in Fig. 15.
